# Association between the oxidative balance score and low muscle mass in middle-aged US adults

**DOI:** 10.3389/fnut.2024.1358231

**Published:** 2024-04-05

**Authors:** Kun Chen, Qiang Yin, Jiangan Guan, Jingwen Yang, Yuan Ma, Yu Hu, Chan Chen, Wenwen Chen

**Affiliations:** ^1^Department of Thoracic Surgery, The First Affiliated Hospital of Wenzhou Medical University, Wenzhou Medical University, Wenzhou, China; ^2^Department of Geriatric Medicine, The First Affiliated Hospital of Wenzhou Medical University, Wenzhou Medical University, Wenzhou, China

**Keywords:** oxidative balance score, oxidative stress, low muscle mass, sarcopenia, diet

## Abstract

**Background:**

Oxidative Balance Score (OBS) is a tool for assessing the oxidative stress-related exposures of diet and lifestyle. The study aimed to investigate the association between OBS and low muscle mass.

**Methods:**

Overall, 6,307 individuals over the age of 18 were assessed using data from the 2011 to 2018 National Health and Nutrition Examination Survey (NHANES). Weighted logistic regression and models were used, together with adjusted models.

**Results:**

There was a negative relationship between OBS and low muscle mass [odds ratio (OR): 0.96, 95% confidence interval (CI): 0.94–0.97, *p***<** 0.0001] using the first OBS level as reference. The values (all 95% CI) were 0.745 (0.527–1.054) for the second level, 0.650 (0.456–0.927) for the third level, and 0.326 (0.206–0.514) for the fourth level (P for trend <0.0001). Independent links with low muscle mass were found for diet and lifestyle factors. A restricted cubic spline model indicated a non-linear association between OBS and low muscle mass risk (P for non-linearity<0.05). In addition, the inflection points of the nonlinear curves for the relationship between OBS and risk of low muscle mass were 20.

**Conclusion:**

OBS and low muscle mass were found to be significantly negatively correlated. By modulating oxidative balance, a healthy lifestyle and antioxidant rich diet could be a preventive strategy for low muscle mass.

## 1 Introduction

Sarcopenia is a common muscle disease associated with aging and is defined as low levels of both muscle mass and strength (1). Sarcopenia is linked with various adverse outcomes including increased risks of falls and fractures (2, 3), resulting in reduced mobility and quality of life. Associations between sarcopenia and various diseases and disorders have been reported, including cardiovascular and respiratory disease, liver cirrhosis, and cognitive impairment (4–8). Additionally, sarcopenia patients have higher risks of hospitalization and higher costs of hospital care, which impose a heavy economic burden on individuals, healthcare systems, and society. Low muscle mass is a major feature of sarcopenia and is also associated with age-related disorders (9). The muscular system is important in maintaining human function. Therefore the early prevention or even reversal of low muscle mass is essential to prevent the progression of sarcopenia and its negative consequences.

Oxidative stress occurs when the balance between oxidants and antioxidants is disrupted, which results from the excessive accumulation of free radicals such as reactive oxygen species (ROS) and reactive nitrogen species (RNS) (10). Oxygen derived free radicals and their derivatives are collectively referred to as ROS, which have oxidative properties. Although the mechanism of skeletal muscle damage has not yet been elucidated, the recent research have explained that the increased production of reactive oxygen species causes a decrease in myoblasts and trigger muscle atrophy apoptosis of muscle cells and may also induce a decrease in muscle strength (11).

Evidence indicates that consumption of nutrients such as vitamin C (12) and vitamin E (13), can prevent oxidative stress, while prooxidants such as smoking (14) and higher iron (15) intake can increase ROS levels and induce oxidative stress-related cell damage. As factors influencing the oxidative balance in individuals may be small but significant, possibly involving multiple interactions between prooxidant and antioxidants (16), a comprehensive assessment of exposure to these factors may more accurately indicate the overall oxidative stress burden in individuals.

The Oxidative Balance Score (OBS) is a composite measure of oxidative stress-related exposures that assesses the oxidative balance of an individual (17). OBS reflects the overall oxidative stress burden by determining both pro- and antioxidant factors in the lifestyle and diet. Lower OBS values reflect higher exposure to oxidants (18). Previous studies have reported associations between OBS and diseases linked to inflammation, such as cardiovascular and chronic kidney disease, type 2 diabetes, and cancer (19–23). However, there has been no previous research on the relationship between OBS and low muscle mass. Therefore, based on the National Health and Nutrition Survey (NHANES) from 2011 to 2018, we performed a cross-sectional analysis to assess the relationship between diet- and lifestyle-associated factors in OBS and low muscle mass.

## 2 Methods

### 2.1 Data and participants

This research used data from the NHANES conducted between 2011 and 2018. NHANES is a survey program by the Centers for Disease Control and Prevention (CDC) aimed at assessing the health and nutritional statuses of individuals in the United States. These were assessed in NHANES using stratification, multiple stages, and probability clusters. The protocols were approved by the Institutional Review Board of the National Center for Health Statistics, with participants providing written informed consent.

**Figure 1:**
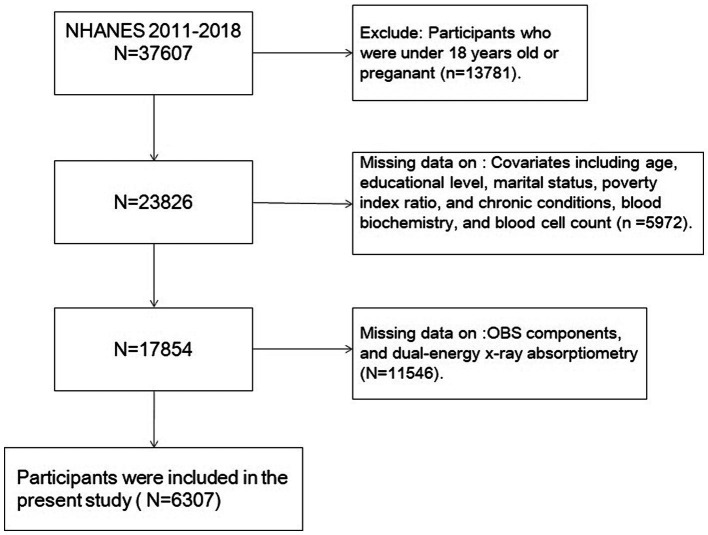
Flowchart portraying the sample selection.

Individuals <18 years were not included, nor were those with missing data. Data on age, sex, race, education information, marital status, income, dual-energy x-ray (DXA) absorptiometry, diet, body mass index (BMI), blood biochemistry, blood cell counts, physical activity, smoking, alcohol consumption, and cotinine were included. Finally, 6,307 eligible samples were included in the analysis (Figure 1).

### 2.2 Assessment of body compositions, DXA, and low muscle mass

The measurements included height in cm, weight in kg, and waist circumference in cm. Pregnant women and those with positive urine test for pregnancy were not included in the study. Additionally, individuals weighing over 136 kg or standing taller than 195.6 cm were not eligible for DXA scanning.

BMI was determined as weight/height^2^. DXA scans were conducted on participants up to the age of 59 using a Hologic Discovery model A densitometer (Hologic, Bedford, MA, United States). Skeletal muscle was defined as mass that was neither fat nor bone, and appendicular skeletal muscle mass (ASM) was determined as the total mass of the lean soft tissue of the extremities. Muscle mass was assessed by the ASM index (ASMI) which was determined as the total ASM in kg divided by the BMI in kg/m^2^ (24). Low muscle mass was assessed using the ASMI with the cut-off values of ASMI <0.512 for women and < 0.789 for men (5).

### 2.3 Exposure definitions

The OBS was classified into dietary and lifestyle OBS. OBS was determined as described (25) by combining 5 prooxidant and 15 antioxidant exposure factors.

The NHANES assessed nutritional intake using a 24-h dietary recall interview. In-person interviews of all participants were conducted by individuals trained in dietary assessment. In addition, the analysis of lifestyle OBS included factors such as BMI, smoking and drinking, and physical activity.

In previous literatures, most OBS used both the population-dependent and predefined components (26–30). The relationship between the outcome variable and OBS can be analyzed using various methods based on the study size and OBS distribution. For example, by dividing OBS into ordinal categories or by using OBS as a continuous variable (31). Regarding to the population-dependent components, they were mostly divided into quantiles or tertiles (26, 27, 32, 33). Consumption of alcohol (predefined components) was assessed by the number of alcoholic drinks taken each day over the previous year. The categories used were non-drinkers and light drinkers, defined as 0–5 g/d for females and 0–30 g/d for males and heavy drinkers (≥15 g/d for females and ≥ 30 g/d for males), which were allocated 2, 1, and 0 points, respectively (25). All continuous score variables were divided into tertiles on the basis of the distribution of these variables in the participants. Then, other components were divided into three groups by their sex-specific tertiles. For dietary antioxidants, the first to third tertile were assigned score of 0–2, while prooxidants were given scores of 0 for the top tertile and 2 for the bottom tertile, as summarized in Table 1. We chose plasma cotinine, the main metabolite of nicotine, for quantitative exposure assessment studies. This was used as cotinine not only has a longer half-life than nicotine in the blood but can also assess “passive smoking,” i.e., exposure to environmental tobacco smoke (ETS). Physical activity was assessed by the metabolic equivalent task (MET), using the formula: physical activity (met·min/week) = recommended MET × exercise time for corresponding activities (min/day) × the number of exercise days per week (day) (34).

**Table 1:** Components that make up the oxidative balance score.

OBS components	Property	Male			Female		
		0	1	2	0	1	2
Dietary OBS components							
Dietary fiber (g/d)	A	<13.55	13.55–22	≥22	<11.45	11.45–18	≥18
Carotene (RE/d)	A	<50.708	50.708–171.91	≥171.91	<55.84	55.84–196	≥196
Riboflavin (mg/d)	A	<1.766	1.766–2.63	≥2.63	<1.378	1.378–1.976	≥1.976
Niacin (mg/d)	A	<24.874	24.874–34.812	≥34.812	<17.095	17.095–24.147	≥24.147
Vitamin B6 (mg/d)	A	<1.841	1.841–2.74	≥2.74	<1.322	1.322–1.962	≥1.962
Total folate (mcg/d)	A	<340.667	340.667–517.833	≥517.833	<260.5	260.5–392.5	≥392.5
Vitamin B12 (mcg/d)	A	<3.61	3.61–6.288	≥6.288	<2.485	2.485–4.335	≥4.335
Vitamin C (mg/d)	A	<39.467	39.467–99.467	≥99.467	<38.317	38.317–87.983	≥87.983
Vitamin E (ATE) (mg/d)	A	<6.687	6.687–10.597	≥10.597	<5.685	5.685–8.79	≥8.79
Calcium (mg/d)	A	<799.167	799.167–1,215	≥11,215	<634	634–956.667	≥956.667
Magnesium (mg/d)	A	<270.5	270.5–384.833	≥384.833	<216	216–297.5	≥297.5
Zinc (mg/d)	A	<10.127	10.127–14.69	≥14.69	<7.282	7.282–10.398	≥10.398
Copper (mg/d)	A	<1.044	1.044–1.516	≥1.516	<0.852	0.852–1.219	≥1.219
Selenium (mcg/d)	A	<110.317	110.317–154.817	≥154.817	<79.017	79.017–112	≥112
Total fat (g/d)	P	≥105.92	73.578–105.92	<73.578	≥79.18	54.952–79.18	<54.952
Iron (mg/d)	P	≥18.407	12.758–18.407	<12.758	≥13.855	9.608–13.855	<9.608
Lifestyle OBS components							
Physical activity (MET-minute/week)	A	<1800	1800–6,480	≥6,480	<1106.667	1106.667–3,360	≥3,360
Alcohol (g/d)	P	≥30	0–30	None	≥15	0–15	None
Body mass index (kg/m2)	P	≥29.8	25.4–29.8	<25.4	≥31.4	24.9–31.4	<24.9
Cotinine (ng/mL)	P	≥0.038	0.022–5.183	<1.13	≥0.096	0.011–0.096	<0.011

A stood in for the antioxidant, P for the pro-oxidant. RE for the retinal equivalent, ATE for the alpha-tocopherol equivalent, and MET for the metabolic equivalent.

In summary, the overall OBS was determined by summing the points for the individual components, with higher OBS values indicating higher antioxidant levels and low pro-oxidant levels.

The OBS had combined the contributions of both diet and lifestyle. To study whether diet or lifestyle factors would have a significant impact on the association between OBS and low muscle mass, respectively, we calculated a dietary OBS by excluding four lifestyle variables: BMI, cotinine, alcohol consumption, and physical activity from the OBS measures that have been described above and calculated a lifestyle OBS that only included these four variables OBS.

### 2.4 Covariates

The demographic variables assessed were age (years), race (Black/White/other race), sex (female/male), marital status (widowed/married/never married/divorced or separated), educational level (high school or above and below high school), and the family income to poverty ratio (PIR) (0–1.0, ≥1 to 2.0, ≥2.0 to 4.0, and ≥ 4.0) (The higher the ratio, the more affluent). Hypertension, diabetes mellitus (DM) and cerebrovascular disease (CVD), asthma, chronic obstructive pulmonary disease (COPD), and chronic kidney disease (CKD) were self-reported after diagnosis by physicians. Hypertension was recorded if the participant was taking antihypertensives or had systolic blood pressure ≥ 140 mmHg or diastolic blood pressure ≥ 90 mmHg. Based on previous literatures, variables such as glycated hemoglobin A1c (HbA1c), alanine aminotransferase (ALT), aspartate aminotransferase (AST), lymphocytes, neutrophils, hemoglobin, and platelets could be confounding factors, so they were also included in the model (35–39).

### 2.5 Statistical analyses

All data were analyzed using the nhanesR package with the NHANES complex weighted sampling design. Treating OBS as continuous in logistic regression may lead to weak odds ratios (OR), we divided participants into four groups based on their total OBS scores and the OBS was converted to a categorical variable by quartile and computed p for trend (17, 40) (Q1 ≤ 14; 14 < Q2 ≤ 20; 20 < Q3 ≤ 26; Q4 > 26). Weighted chi-squared tests and linear regression models were used for inter-group comparisons and analysis of variables, categorical and continuous, respectively. Associations between OBS and low muscle mass were assessed by multiple logistic regression models, using unadjusted (Model 1) and age- and sex-adjusted (Model 2) models, as well as Model 3 which was constructed using age, marital status, race, gender, DM, hypertension, CVD, CKD, COPD, asthma, educational status, creatinine, HbA1c, ALT, AST, lymphocytes, neutrophils, hemoglobin, platelets, and PIR. We investigated whether the shape of the relationship between OBS and low muscle mass was non-linear using the restricted cubic spline regression model, and OBS was included in the model as a continuous variable. Finally, stratified and sensitivity analyses were performed to test the consistency of the findings across subgroups and the stability of the results. *p*-values less than 0.05 were considered statistically significant.

## 3 Results

### 3.1 Participant characteristics

The baseline features of the 6,307 participants are shown in Table 2. All participants were Americans aged 20–59 years (average, 39.33 ± 0.35) and included 2,434 (38.59%) White and 3,197 (50.69%) male participants.

**Table 2:** Characteristics of the study population based on oxidative balance score quartiles.

Variable	Total	Q1	Q2	Q3	Q4	*p* value
Age (years)	39.325(0.352)	38.588(0.423)	39.564(0.441)	39.703(0.536)	39.398(0.494)	0.243
Race/ethnicity, n (%)						< 0.0001
Black	1,287(20.406)	494(16.098)	343(11.092)	251(7.238)	199(6.798)	
Other	2,586(41.002)	575(22.181)	638(24.420)	740(25.075)	633(25.857)	
White	2,434(38.592)	636(61.721)	587(64.488)	674(67.686)	537(67.345)	
Education, n (%)						< 0.0001
Less than high school	903(14.317)	308(14.086)	240(11.245)	192(7.307)	163(7.484)	
High school	1,316(20.866)	460(28.773)	313(21.033)	331(19.445)	212(12.855)	
More than high school	4,088(64.817)	937(57.141)	1,015(67.723)	1,142(73.248)	994(79.661)	
Sex, n (%)						0.747
Female	3,110(49.31)	847(48.769)	750(47.233)	824(48.742)	689(49.956)	
Male	3,197(50.69)	858(51.231)	818(52.767)	841(51.258)	680(50.044)	
PIR, n (%)						< 0.0001
0–1.0	1,284(20.358)	439(19.989)	307(13.444)	305(11.951)	233(11.158)	
1.0 ≤ PIR <2.0	1,528(24.227)	492(24.742)	372(17.969)	372(17.347)	292(15.778)	
2.0 ≤ PIR <4.0	1717(27.224)	436(26.973)	444(31.549)	463(30.865)	374(26.437)	
≥4.0	1778(28.191)	338(28.296)	445(37.038)	525(39.837)	470(46.627)	
Marital status, n (%)						< 0.0001
Divorced	571(9.053)	193(11.210)	124(8.210)	148(9.460)	106(7.767)	
Living with partner	678(10.75)	191(10.743)	177(11.353)	182(10.647)	128(7.555)	
Married	3,107(49.263)	722(45.142)	802(55.532)	830(52.773)	753(60.062)	
Never married	1,667(26.431)	497(27.101)	404(21.613)	429(23.893)	337(22.579)	
Separated	203(3.219)	72(4.122)	45(2.424)	53(2.243)	33(1.366)	
Widowed	81(1.284)	30(1.681)	16(0.868)	23(0.984)	12(0.672)	
Low muscle mass, n (%)						< 0.0001
No	5,850(92.754)	1,531(91.176)	1,453(93.986)	1,555(94.691)	1,311(97.358)	
Yes	457(7.246)	174(8.824)	115(6.014)	110(5.309)	58(2.642)	
HbA1c (mmol/L)	5.479(0.015)	5.542(0.028)	5.485(0.029)	5.489(0.024)	5.392(0.024)	< 0.001
Alt (IU/L)	25.775(0.292)	25.255(0.622)	27.176(0.763)	25.607(0.546)	25.034(0.545)	0.156
Ast (IU/L)	25.223(0.264)	24.707(0.664)	26.124(0.678)	24.753(0.398)	25.382(0.505)	0.28
Creatinine (mg/dl)	0.862(0.004)	0.876(0.009)	0.874(0.009)	0.847(0.007)	0.851(0.006)	0.026
Lymphocytes (k/μL)	2.198(0.017)	2.271(0.026)	2.238(0.030)	2.148(0.024)	2.137(0.027)	< 0.001
Neutrophils (k/μL)	4.268(0.038)	4.535(0.067)	4.257(0.056)	4.179(0.047)	4.104(0.057)	< 0.0001
Hemoglobin (g/dl)	14.321(0.033)	14.339(0.061)	14.427(0.051)	14.248(0.044)	14.279(0.043)	0.032
Platelets (k/μL)	240.589(1.250)	245.586(2.184)	238.290(1.816)	242.183(1.993)	235.757(1.866)	0.003
CKD, n (%)						0.001
No	5,786(91.739)	1,533(90.328)	1,435(93.113)	1,548(94.494)	1,270(93.501)	
Yes	521(8.261)	172(9.672)	133(6.887)	117(5.506)	99(6.499)	
COPD, n (%)						0.005
No	6,193(98.192)	1,652(96.612)	1,551(99.096)	1,638(97.989)	1,352(98.348)	
Yes	114(1.808)	53(3.388)	17(0.904)	27(2.011)	17(1.652)	
Hypertension, n (%)						0.006
No	4,595(72.856)	1,162(70.702)	1,140(73.962)	1,241(75.759)	1,052(77.463)	
Yes	1712(27.144)	543(29.298)	428(26.038)	424(24.241)	317(22.537)	
Asthma, n (%)						0.209
No	5,337(84.62)	1,397(83.172)	1,341(85.980)	1,435(85.923)	1,164(83.840)	
Yes	970(15.38)	308(16.828)	227(14.020)	230(14.077)	205(16.160)	
Diabetes mellitus, n (%)						< 0.001
DM	646(10.243)	219(9.983)	176(8.705)	151(7.881)	100(4.660)	
IFG	260(4.122)	76(4.052)	70(4.868)	73(4.105)	41(2.481)	
IGT	159(2.521)	39(1.807)	48(3.137)	39(1.979)	33(1.680)	
No	5,242(83.114)	1,371(84.158)	1,274(83.290)	1,402(86.035)	1,195(91.179)	
Cardiovascular disease, n (%)						0.055
No	6,108(96.845)	1,628(96.438)	1,518(97.133)	1,621(97.710)	1,341(98.348)	
Yes	199(3.155)	77(3.562)	50(2.867)	44(2.290)	28(1.652)	

PIR, the ratio of family income to poverty; COPD, chronic obstructive pulmonary disease; CKD, chronic kidney disease; DM, diabetes mellitus; IFG, impaired fasting glucose; IGT, impaired glucose tolerance; HbA1c, glycated hemoglobin A1c; ALT, alanine aminotransferase; AST, aspartate aminotransferase. Q1:OBS ≤ 14; Q2:14 < OBS ≤ 20; Q3:20 < OBS ≤ 26; Q4: OBS > 26.

Table 2 provides the weighted characteristics of the participants according to the OBS quartiles (Q1 ≤ 14; 14 < Q2 ≤ 20; 20 < Q3 ≤ 26; Q4 > 26). Significant differences in confounders were observed between the different quartiles with participants in the top quartile more likely to be married and to have high PIR. The frequencies of low muscle mass, hypertension, diabetes, CKD, and COPD tended to increase with reduced OBS. Furthermore, in terms of OBS, individuals in the top quartile (Q4 > 26) tended to have higher levels of education.

### 3.2 Relationship between OBS and low muscle mass

As seen in Table 3, in adjusted model 3, higher OBS values were linked with a lower risk of lower muscle mass, with an OR of 0.96 (95% confidence interval [CI] 0.94–0.97, *p* **<** 0.0001).

**Table 3:** Association of oxidative balance score (OBS) with low muscle mass.

OBS	Q1	Q2	Q3	Q4	P for trend
OR (95% CI)					
Model I	Ref	0.661(0.486,0.899)	0.579(0.434,0.773)	0.280(0.184,0.426)	<0.0001
Model II	Ref	0.638(0.464,0.877)	0.559(0.419,0.745)	0.273(0.177,0.419)	<0.0001
Model III	Ref	0.745(0.527,1.054)	0.650(0.456,0.927)	0.326(0.206,0.514)	<0.0001

PIR, the ratio of family income to poverty; COPD, chronic obstructive pulmonary disease; CI, confidence interval; CKD, chronic kidney disease; DM, diabetes mellitus; IFG, impaired fasting glucose; IGT, impaired glucose tolerance; HbA1c, glycated hemoglobin A1c; ALT, alanine aminotransferase; AST, aspartate aminotransferase. Model 1: unadjusted. Model 2: adjusted for age and gender. Model 3: Adjusted for age, gender, race, marital status, hypertension, DM, CVD, CKD, COPD, asthma, education status, HbA1c, creatinine, ALT, AST, lymphocytes, neutrophils, hemoglobin, platelets, and PIR. Q1:OBS ≤ 14; Q2:14 < OBS ≤ 20; Q3:20 < OBS ≤ 26; Q4: OBS > 26.

In this model, using the first OBS level as the reference, it was found that the OR of the second OBS level was 0.745 (95%CI, 0.527–1.054), while that of the third OBS level was 0.650 (95%CI, 0.456–0.927), and that of the fourth OBS level was 0.326 (95%CI, 0.206–0.514) with P for trend<0.0001. Thus at an OBS value >26, the likelihood of low muscle mass was reduced by 67%.

**Figure 2:**
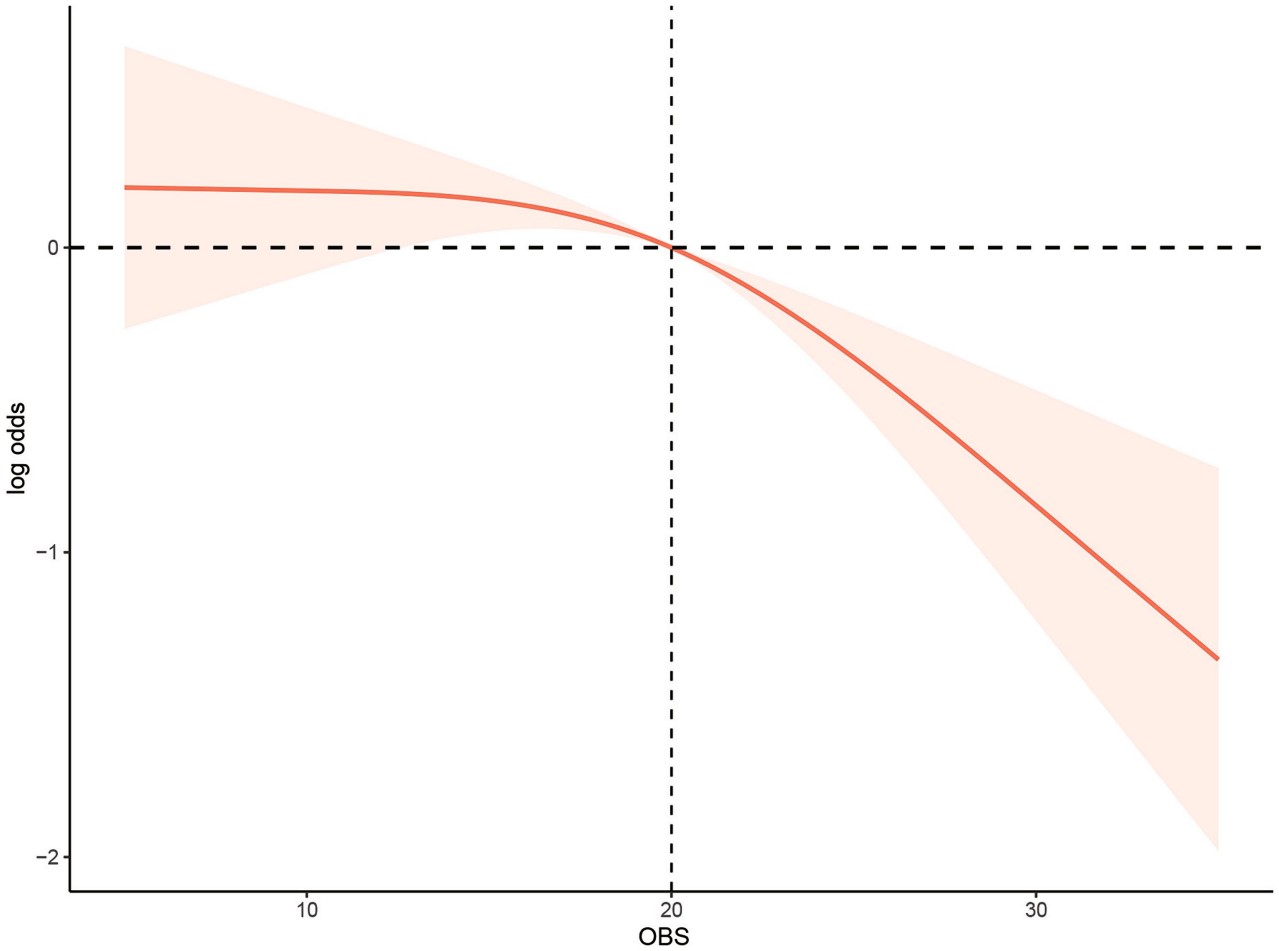
Multivariable-adjusted restricted cubic spline curve for the association between oxidative balance score and the risk of low muscle mass. The solid red line represents the fitted curve; the light red area represents the confidence interval.

The non-linear relationship between the OBS and risk of low muscle mass was assessed using a restricted cubic spline model (P for non-linearity <0.05; Figure 2). In addition, the inflection points of the nonlinear curves for the relationship between OBS and risk of low muscle mass were 20. After the inflection point, OBS was more significantly associated with a reduced odd of low muscle mass, suggesting that OBS were associated with a more significant reduction in odd of low muscle mass after exceeding 20 points.

### 3.3 Relationship between the dietary/lifestyle OBS and low muscle mass

The OBS was classified into dietary- and lifestyle-associated OBS for further evaluation of the associations between these and low muscle mass (Table 4).

**Table 4:** Association of dietary/lifestyle oxidative balance score (OBS) with low muscle mass.

Dietary OBS	OR (95% CI)	*P*-value	Lifestyle OBS, OR (95% CI)	*P*-value
Model I	0.960(0.944,0.976)	<0.0001	0.695(0.645,0.749)	<0.0001
Model II	0.957(0.942,0.973)	<0.0001	0.698(0.648,0.752)	<0.0001
Model III	0.967(0.949,0.986)	0.001	0.746(0.676,0.823)	<0.0001

PIR, the ratio of family income to poverty; COPD, chronic obstructive pulmonary disease; CI, confidence interval; CKD, chronic kidney disease; DM, diabetes mellitus; IFG, impaired fasting glucose; IGT, impaired glucose tolerance; HbA1c, glycated hemoglobin A1c; ALT, alanine aminotransferase; AST, aspartate aminotransferase. Model 1: unadjusted. Model 2: adjusted for age and gender. Model 3: Adjusted for age, gender, race, marital status, hypertension, DM, CVD, CKD, COPD, asthma, education status, HbA1c, creatinine, ALT, AST, lymphocytes, neutrophils, hemoglobin, platelets, and PIR.

Adjusted Model 3 indicated that both dietary- and lifestyle-associated OBS were negatively linked with low muscle mass (OR: 0.967; 95% CI: 0.949–0.986; *p* = 0.001, OR: 0.746; 95% CI: 0.676–0.823; *p* < 0.0001, respectively).

### 3.4 Sensitivity analysis

Adjustment of the full model indicated relationships between OBS and low muscle mass in terms of PIR (*p* = 0.022), with higher OBS predicted to reduce the risk of low muscle mass in people with PIR of 0–1, 1–2, and greater than 4 (OR: 0.954, 95% CI: 0.925–0.984; OR: 0.965, 95% CI: 0.941–0.991; OR: 0.908, 95% CI: 0.866–0.953, respectively), while OBS was less likely to influence the risk of low muscle mass in individuals with PIR of 2.0–4.0 (OR: 0.969, 95% CI: 0.937–1.001) (Table 5). OBS and low muscle mass were observed to be significantly associated in people with educational levels of high school and above (P for interaction was 0.018) (OR: 0.970, 95% CI: 0.941–0.999; OR: 0.939, 95% CI: 0.913–0.967, respectively), but less associated in those with educational levels below high school. The remaining subgroups showed no significant associations (*p* > 0.05).

**Table 5:** Association between oxidative balance score and low muscle mass in subgroups.

Variable	Adjust OR (95% CI)	P	P for interaction
Age (years)			0.394
<40	0.964(0.935, 0.993)	0.016	
≥40	0.951(0.927, 0.976)	<0.001	
Race/ethnicity			0.072
Black	0.903(0.846, 0.964)	0.004	
Other	0.961(0.936, 0.986)	0.003	
White	0.949(0.925,0.975)	<0.001	
Education			0.018
Less than high school	0.968(0.930, 1.008)	0.110	
High school	0.970(0.941, 0.999)	0.044	
More than high school	0.939(0.913, 0.967)	<0.0001	
Sex			0.893
Female	0.953(0.924,0.983)	0.003	
Male	0.960(0.940, 0.980)	<0.001	
PIR			0.022
0–1.0	0.954(0.925, 0.984)	0.004	
1.0 ≤ PIR <2.0	0.965(0.941,0.991)	0.009	
2.0 ≤ PIR <4.0	0.969(0.937, 1.001)	0.060	
≥4.0	0.908(0.866, 0.953)	<0.001	
Marital status, n (%)			0.303
Divorced	0.986(0.940, 1.033)	0.532	
Living with partner	0.963(0.920, 1.009)	0.111	
Married	0.950(0.926, 0.976)	<0.001	
Never married	0.927(0.890, 0.965)	<0.001	
Separated	0.941(0.877, 1.009)	0.084	
Widowed	0.998(0.834,1.194)	0.983	
CKD			0.843
No	0.955(0.937,0.972)	<0.0001	
Yes	0.959(0.910, 1.010)	0.109	
COPD			0.119
No	0.959(0.942,0.976)	<0.0001	
Yes	0.754 (0.601,0.946)	0.018	
Hypertension			0.105
No	0.967(0.946, 0.987)	0.002	
Yes	0.939(0.912, 0.966)	<0.0001	
Asthma			0.186
No	0.960(0.943, 0.977)	<0.0001	
Yes	0.938(0.888, 0.990)	0.021	
Diabetes mellitus			0.639
No	0.952(0.934,0.972)	<0.0001	
IGT	0.856 (0.672,1.090)	0.176	
DM	0.966(0.916, 1.018)	0.188	
IFG	0.974(0.902, 1.052)	0.484	
Cardiovascular disease			0.123
No	0.960(0.942,0.979)	<0.001	
Yes	0.840(0.754, 0.935)	0.004	

PIR, the ratio of family income to poverty; COPD, chronic obstructive pulmonary disease; CI, confidence interval; CKD, chronic kidney disease; DM, diabetes mellitus; IFG, impaired fasting glucose; IGT, impaired glucose tolerance; HbA1c, glycated hemoglobin A1c; ALT, alanine aminotransferase; AST, aspartate aminotransferase. Adjusted for age, gender, race, marital status, hypertension, DM, CVD, CKD, COPD, asthma, education status, HbA1c, creatinine, ALT, AST, lymphocytes, neutrophils, hemoglobin, platelets, and PIR.

## 4 Discussion

This cross-sectional study used NHANES data from 2011 to 2018, resulting in the enrollment of 6,307 individuals meeting the inclusion criteria. OBS was used to represent the degree of oxidative stress and investigated the link between OBS and low muscle mass. The univariate logistic regression model indicated a negative association between the two. Following adjustment of confounders, it was still found that OBS was independently linked with the increased odds of low muscle mass. The maximum level of OBS was found to be 36 and did not increase indefinitely. Over the range of 0–36, higher OBS values reflected lower odds of low muscle mass. In addition, dietary and lifestyle components were associated with low muscle mass independently. Our results demonstrate the value of antioxidant-rich diets and lifestyles in improving muscle mass.

The findings on OBS and low muscle mass are reliable and similar to earlier investigations. The presence of oxidative stress is a possible reason for the link between OBS and low muscle mass, leading to disrupted oxidative homeostasis in the body with the production of excess ROS and RNS. ROS accumulation may induce atrophy of skeletal muscles possibly exacerbated by oxidative stress associated with chronic diseases. As shown by Zhang et al., conditions such as aging, obesity, and cancer can increase ROS generation in muscles (41), potentially inducing oxidative damage and the disruption of mitochondrial functioning. This can lead to reduced ATP generation and protein synthesis, as well as protein degradation, contributing to reduced muscle mass and dysfunction (42, 43). In the dietary OBS section, higher OBS scores often mean more intake of antioxidant components. Many dietary components of OBS have been believed to inhibit oxidative stress and contribute to the management of sarcopenia (44). Both vitamin C and vitamin E can function as ROS scavengers and can thus mitigate oxidative stress. Vitamin E is known to promote the repair of membrane injury in skeletal muscle cells (45). A study showed that increased vitamin E intake enhanced muscle mass (46) while other studies indicated that vitamin C supplementation reduced oxidative stress, and that increased dietary intake of the vitamin C alleviated muscle loss in younger women (47, 48). Besides, in skeletal muscle physiology, vitamin C helps with carnitine and collagen production and has a positive association with muscle (49). It is reported that sarcopenia patients tend to have reduced intake of minerals such as calcium, selenium, and magnesium relative to healthy individuals (50). However, some research also suggested that certain antioxidant supplements like vitamin E and C might impair adaptations to resistance training (51, 52). Therefore, special caution should be taken with these supplements during endurance training. Nonetheless, the effects of antioxidants on muscle mass/strength might also depend on the individual’s oxidative stress/antioxidant balance (53).

The link between lifestyle and low muscle mass has been demonstrated. Multiple studies have shown that smoking is a risk factor for skeletal muscle dysfunction (54, 55). Smoking causes skeletal muscle dysfunction by reducing oxygen delivery to the mitochondria, which can lead to chronic muscle atrophy and sarcopenia (56). In addition, alcohol can also increase the incidence of low hand grip strength (57). Alcohol consumption may disrupt protein metabolism in skeletal muscles and response to anabolic stimuli by reducing overall protein synthesis (58). Additionally, exercise can help reduce mitochondrial damage caused by aging by inhibiting oxidative stress, DNA damage, and apoptosis in mitochondria (59).

Consideration of a single factor only may be insufficient to explain its antioxidant effect. As a comprehensive indicator of oxidative/antioxidant balance, the OBS may provide a better reflection of the overall state of oxidative stress than any individual factors considered alone.

We then further investigated the nonlinear relationship between OBS and odd of low muscle mass and found that there was the J-shaped relationship between OBS and odd of low muscle mass. The inflection points for OBS were 20 points. Before the inflection point, increasing OBS hardly affected the odd of low muscle mass, but after the inflection point, increasing OBS would have a greater impact on the odd of low muscle mass. This may be due to: After reaching this balance point, the antioxidant capacity exceeded its oxidation capacity in the individual oxidation balance system (30, 60). Earlier investigations have reported significant links between socioeconomic status (including educational levels and income) and sarcopenia (61). The current research findings are consistent with this. It was found that the participants with higher OBS tended to have higher PIR and education. Usually, a higher level of education often leads to more employment opportunities and higher income and contributes to healthier lifestyles and behaviors. Educational level was found to be a factor in translating nutritional knowledge into improved dietary habits, as indicated by a study on diet quality in the United States (62). Participants with higher levels of education tended to consume more fruit, vegetables, and whole grains, while controlling their intake of solid fats, alcohol, and added sugar (63).

The study had several limitations. First, due to its cross-sectional design, it is possible that there may have been additional confounders; furthermore, causal associations could not be determined. Second, the use of self-reported recall in the NHANES data may have introduced both recall and reporting bias. Lastly, while the correlation found between muscle mass and OBS is significant, it is not very substantial. This is likely due to the population being used. DXA scans were only used to assess participants aged between 8 and 59 years and thus the study did not include older participants. Because loss of muscle mass usually starts in 4th decade of life and the average age of participants was 39 years old in our research (64), the low muscle mass in elderly people is more than in young people. This affects the generalization of the results and introduces selection bias. Prospective cohort studies and studies involving older adults are thus needed in the future.

## 5 Conclusion

It was found that OBS and low muscle mass were significantly negatively correlated. Nevertheless, further randomized controlled trials are needed to assess the causal connection. These findings may be useful for the stratification of low muscle mass risk in the general population, allowing timely interventions.

## Data availability statement

The original contributions presented in the study are included in the article/supplementary material, further inquiries can be directed to the corresponding authors.

## Ethics statement

The studies involving humans were approved by the Institutional Review Board of the National Center for Health Statistics. The studies were conducted in accordance with the local legislation and institutional requirements. The participants provided their written informed consent to participate in this study.

## Author contributions

KC: Conceptualization, Data curation, Formal analysis, Funding acquisition, Investigation, Methodology, Project administration, Resources, Software, Supervision, Validation, Visualization, Writing – original draft, Writing – review & editing. QY: Writing – original draft, Writing – review & editing. JG: Writing – original draft, Writing – review & editing. JY: Writing – original draft, Writing – review & editing. YM: Writing – original draft, Writing – review & editing. YH: Writing – original draft, Writing – review & editing. CC: Conceptualization, Data curation, Investigation, Software, Writing – original draft, Writing – review & editing. WC: Conceptualization, Data curation, Formal analysis, Funding acquisition, Investigation, Methodology, Project administration, Resources, Software, Supervision, Validation, Visualization, Writing – original draft, Writing – review & editing.
